# A case report of severe *Fusobacterium nucleatum* sepsis secondary to nephrectomy

**DOI:** 10.1186/s12879-022-07294-6

**Published:** 2022-03-29

**Authors:** Chang Liu, Qiming Jia, Lifeng Wang, Dong Yang

**Affiliations:** 1 Critical Care Medicine, Affiliated Tumor Hospital of Zhengzhou University, Henan Cancer Hospital, Zhengzhou, 450008 Henan China; 2 Department of Diagnostic Radiology, Affiliated Tumor Hospital of Zhengzhou University, Henan Cancer Hospital, Zhengzhou, Henan China; 3 Department of Urology, Affiliated Tumor Hospital of Zhengzhou University, Henan Cancer Hospital, Zhengzhou, Henan China

**Keywords:** *Fusobacterium nucleatum*, Kidney cancer, Renal vein thrombosis, Sepsis

## Abstract

**Background:**

*Fusobacterium nucleatum (F. nucleatum)* is a resident anaerobic bacterium, which in rare cases may invade blood from the head and neck or the digestive tract to cause bacteremia and induce venous thrombosis. *F. nucleatum* is closely related to abdominal tumors, but it has not been reported in relation to renal tumors. We report herein a possible case.

**Case presentation:**

This patient had kidney cancer with thrombosis in the right renal vein but had no sign of infection. After radical nephrectomy, thrombi formed in his left renal vein, and when removed, severe sepsis occurred. He did not respond to treatment with antibiotics and died, but the blood culture done confirmed that he had *F. nucleatum* bacteremia.

**Conclusion:**

*F. nucleatum* may also be associated with kidney cancer, and could cause post-operative renal vein thrombosis, and sepsis or septic shock after thrombectomy.

## Background

*Fusobacterium nucleatum (F. nucleatum)* is a gram-negative anaerobe that exists in the upper respiratory tract, gastrointestinal tract, and female urogenital tract [1]. It is an opportunistic pathogen. The first case reported with bacteremia followed suppurative thrombophlebitis of the internal jugular vein associated with oropharyngeal infection [2]. This suggested that thrombosis may be a complication of bacterial infection. Subsequently, thrombosis was reported in other veins, such as portal vein [3], hepatic vein [4], inferior vena cava [5], and dural venous sinus [6], but renal vein thrombosis (RVT) has been reported in only one case [7]. *F. nucleatum* is often associated with abdominal tumors, such as rectal and ovarian cancers [8], but there is no report of its association with kidney cancers. We report the case of a patient who had RVT after radical nephrectomy and developed severe sepsis due to the spread of *F. nucleatum* following thrombectomy. Whether kidney cancer is also associated with *F. nucleatum* infection, thereby increasing the risk of thrombectomy, is an issue that clinicians, particularly surgeons, would need to be aware of.

## Case presentation

**Fig. 1:**
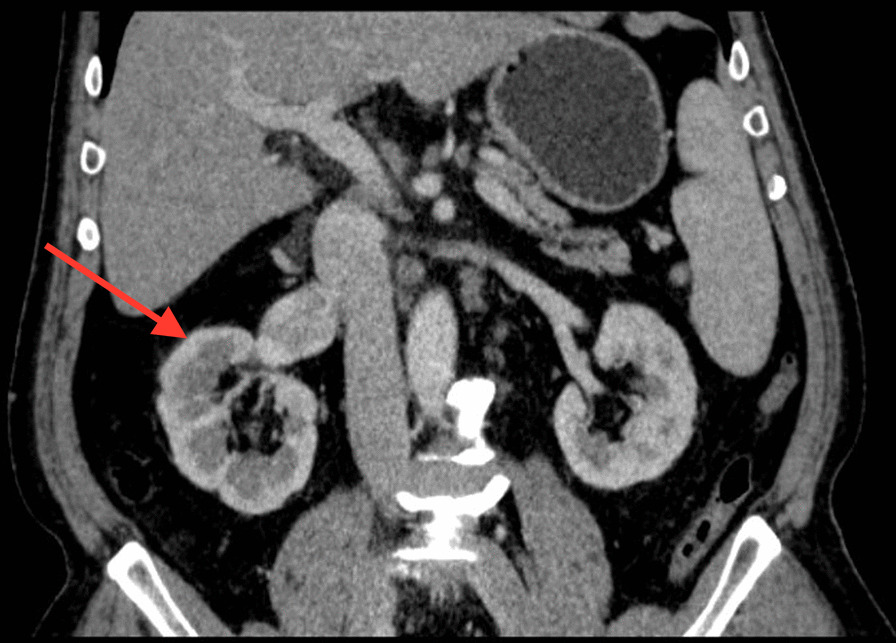
CT showed a mass in the right kidney. The right kidney was significantly enlarged, and the right renal vein was significantly thickened

**Fig. 2:**
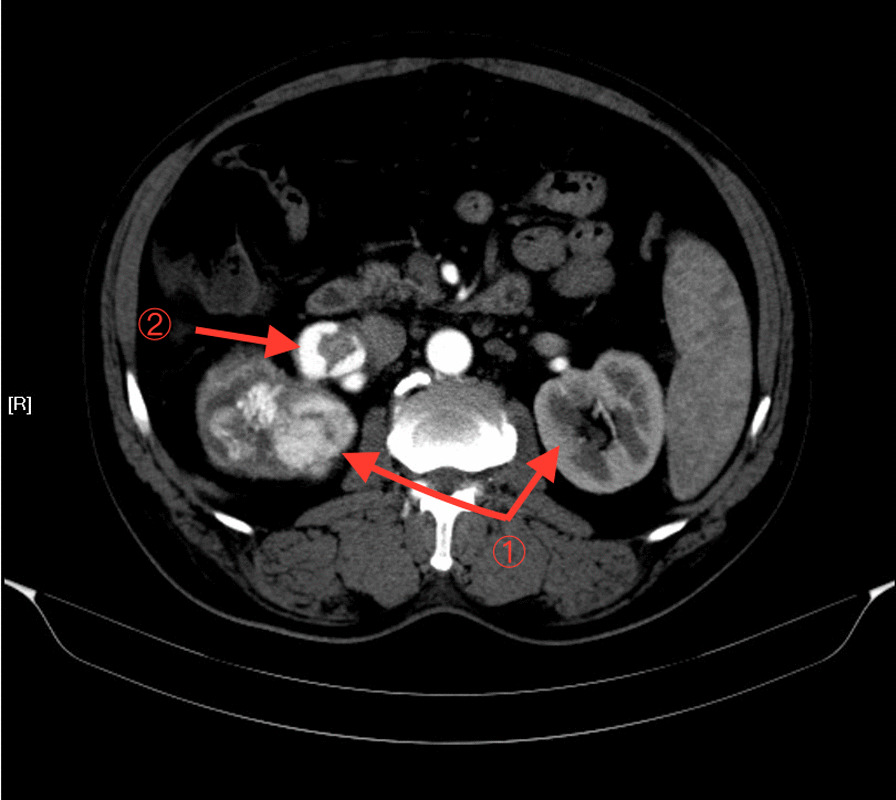
CTA showed that ① the mass in the right kidney was significantly enhanced, but that in the left kidney was not significantly enhanced; ② there was thrombosis in the right renal vein, while the left renal vein had no thrombosis

**Fig. 3:**
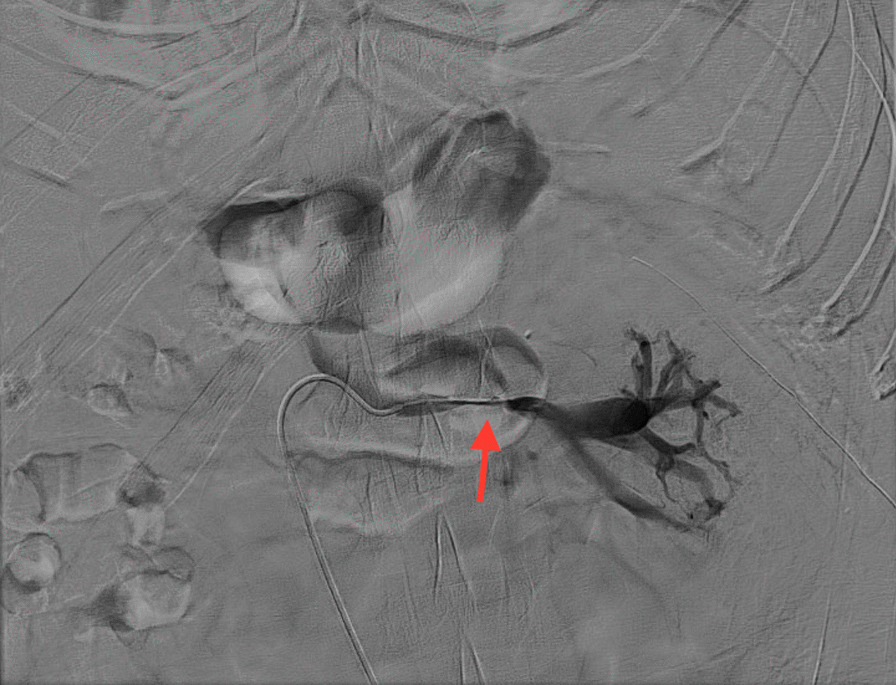
Selective angiography showed a 2 cm filling defect at the initiation site of the left renal vein

This Chinese patient was 59 years old. He was healthy before, without history of thrombosis. When he was admitted to our hospital, CT examination showed a mass in the right kidney, and there was no sign of infection. However, the subsequent CT angiography showed that the mass in the right kidney had abundant blood supply, and there was thrombosis in the right renal vein. Near the left renal vein there was another mass, but there were no thrombi in the left renal vein and portal vein (Figs. 1 and 2). The entire right kidney and the mass in the left kidney were excised by surgeons, and histological examination suggested WHO/ISUP grade-3 clear cell carcinoma. Post-operatively, he developed acute kidney injury (AKI) as evidenced by decreased urine volume (0.27 ml/h/kg for 3 h) and increased serum creatinine (75 mol/l higher than the preoperative level), and selective left renal venography showed a 2 cm filling defect in the left renal vein, suggesting thrombosis (Fig. 3). After the thrombus was removed, we performed continuous venovenous hemodiafiltration (CVVHDF) on the patient. Within the first 12 h, the patient was conscious, with stable vital signs. In addition, he had no fever, and the urine volume exceeded 40 ml/h, indicating that AKI was prerenal AKI caused by the thrombus in his left renal vein. Thus, he improved quickly after thrombectomy.

However, in the following 12 h, the patient showed signs of infection. His consciousness became poor, while body temperature and heart rate increased, and blood pressure, urine volume and oxygenation index decreased. The high CRP (71.82 mg/l) and PCT (22.82 ng/ml) levels also suggested that the patient might be infected. Based on the 2016 SSC guidelines, the patient had septic shock. In addition, SOFA score and laboratory test results were deteriorating (Table 1). Therefore, we immediately started fluid resuscitation, drew the patient's blood for culture, and empirically commenced meropenem and teicoplanin for treatment of the suspected sepsis.

**Table 1:** SOFA score and laboratory test results post-thrombectomy

Time post-thrombectomy (h)	LEU (10^9/l)	NEU %	OI (mmHg)	PH	BE (mmol/l)	LAC (mmol/l)	SOFA
12	10.65	74.9	342	7.278	− 3.9	1.8	5
12–24	9.64	83.1	152	7.303	− 5.1	3.7	13
24–48	11.87	87.6	63	7.173	− 13.8	9.5	20

*LEU* leukocyte count, *NEU* neutrophil ratio, *OI* oxygen index, *BE* base excess, *LAC* lactic acid

Unfortunately, he continued to deteriorate such that by the second day his respiratory and circulatory systems collapsed and he required ventilation with almost pure oxygen. He died on the third day of respiratory and circulatory failure, and the result of the blood culture, which was received two days later, showed that he had *F. nucleatum* bacteremia, sensitive to penicillin, cefoxitin, piperacillin/tazobactam, cefoperazone/sulbactam, imipenem/cilastatin, meropenem, clindamycin and metronidazole, intermediate to ceftriaxone, and resistant to none.

## Discussion and conclusion

This was a rapidly progressive case of septic shock due to *F. nucleatum*. The patient had no signs of infection before the operation, and full aseptic precautions were observed in all the operations. Therefore, we speculate that the *F. nucleatum* infection was associated with the patient's kidney cancer, as suggested by thrombosis in the right renal vein. The subsequent sepsis and thrombosis in the left renal vein were accompanied by *F. nucleatum* bacteremia.

The original source of the infection is unknown, but the reported sources are mainly concentrated in the head and neck and the abdominal cavity [9], although the bacteria from these sources have not been reported to cause RVT. Forming venous thrombosis after blood stream invasion is the prerequisite for *F. nucleatum to* disseminate septic emboli, because it promotes the aggregation of platelets [10].

Each year, only 5.5 to 7.6 people out of 1,000,000 develop *F. nucleatum* bacteremia, but the mortality rate is as high as 10–15% [6, 11]. Compared with women, men are more prone to *F. nucleatum* bacteremia, and the mortality rate of patients above 40 years old is much higher [12, 13]. Tumors also increase the risk of infection [14]. The main cause of death is the dissemination of septic emboli and the formation of abscesses in special parts [15].

Our patient was male and over 40 years old. The kidney cancer may have compromised his local immunity, and subsequently *F. nucleatum* invaded the blood to form a thrombus in the renal vein. There is no evidence that the bacterium is from the head and neck or the abdominal cavity, but the thrombus in the renal vein suggests that the bacterium might have originated from the urinary tract.

*F. nucleatum* is closely related to abdominal tumors and its detection rate is highest in patients with colorectal cancer [16]. However, the mechanism by which it induces tumor formation is unknown [17]. There are no reports about the association of *F. nucleatum* with kidney cancer (Table 2), but we speculate that the bacterium could also be associated with the occurrence of renal tumors. Combining the 13 cases in Table 2 and the 22 cases collected by Yusuf et al. [18], we summarize the characteristics of the 35 cases reported so far in Table 3.

**Table 2:** Case of *F. nucleatum* infection

Case/patient	Age range (year)	Sex	Background	Presentation	Source	Thrombosis	Treatment	Susceptibility	Treatment duration	Outcome
1	40–50	Male	Rectal cancer	Fever, lower abdominal pain	Bowel perforation with intra-abdominal abscess	None	Drainage + meropenem + vancomycin then amoxicillin/clavulanate	Meropenem (S) Amoxicillin/clavulanate (N/T) but penicillin (S)	28 days	Survived
2	90–100	Female	AF, HTN	Fever, lethargy	Mass in the posterior oral cavity (biopsy: squamous cell carcinoma), liver abscess	None	Piperacillin/tazobactam	S	1 day	Died
3	40–50	Male	Sickle cell anemia	No fever, lower limb pain	Unknown	Deep vein thrombosis	Anti-coagulation	N/T	NA	Survived
4	20–30	Male	Developmental delay, deafness, seizure disorder	Fever, cough, shortness of breath, vomiting	Liver abscess	None	Vancomycin + ceftriaxone then piperacillin/tazobactam + metronidazole + drainage	NA	> 6 weeks	Survived
5	20–30	Male	Sickle cell anemia	Fever, flank pain	Unknown	Left renal vein thrombosis	Anti-coagulation + metronidazole then amoxicillin/clavulanate	Metronidazole(N/T) Amoxicillin/clavulanate(N/T) but meropenem(S) and penicillin(S)	28 days	Survived
6	20–30	Male	None	Fever, right upper quadrant abdominal pain	Liver abscess	None	Vancomycin + piperacillin/tazobactam + drainage then ertapenem	NA	> 10 weeks	Survived
7	70–80	Female	Ovarian cancer, HTN, CAD	No fever, cough	Lower respiratory tract	None	Meropenem + moxifloxacin	Meropenem(S) Moxifloxacin(N/T)	14 days	Died
8	70–80	Male	HTN, DM, CAD	Fever, dull, epigastric abdominal pain	Unknown	Hepatic vein thrombosis	Cefepime then anti-coagulation + clindamycin	Clindamycin(S) Cefepime(N/T) but cefoxitin(S)	> 14 days	Survived
9	40–50	Male	Chronic pancreatitis and pancreatic pseudocyst	Fever, myalgias	Liver abscess	Hepatic vein thrombosis	Ceftriaxone + ofloxacin then amoxicillin/clavulanate + metronidazole + anti-coagulation + drainage	Ceftriaxone(N/T) Ofloxacin(N/T) Amoxicillin/clavulanate(S) Metronidazole(S)	10 months	Survived
10	20–30	Male	None	Fever, sore throat, right neck pain and chest pain	Acute tonsillitis, pyothorax	Right internal jugular vein thrombophlebitis	Ampicillin/sulbactam then penicillin G then clindamycin then amoxicillin/clavulanate	NA	6 weeks	Survived
11	40–50	Male	Dementia, epilepsy	Fever, pain over right hip	Hip abscess	None	Ampicillin/sulbactam + Fosfomycin + metronidazole + surgery then amoxicillin/clavulanate + metronidazole	Metronidazole(S) Ampicillin/sulbactam(N/T) Amoxicillin/clavulanate(N/T) but penicillin(S)	6 weeks	Survived
12	10–20	Male	Gonorrhea	Fever, sore throat, cough and chest pain	Tonsillitis, pneumonia	Right internal jugular vein thrombophlebitis	Levofloxacin then ampicillin-sulbactam then ampicillin then metronidazole	NA	> 7 days Discharged with 4 weeks' metronidazole	Unknown (lost)
13	60–70	Female	Ovarian cancer	Fever, abdominal pain	Intra-abdominal	None	NA	NA	2 days	Died

*AF* atrial fibrillation, *HTN* hypertension, *CAD* coronary artery disease, *DM* diabetes mellitus, *NA* not-applicable, *S* sensitive, N/T: Not-tested. Case 1, 3, 5, 7 [7], case 2 [20], case 4 [21], case 6 [22], case 8 [4], case 9 [23], case 10 [24], case 11 [25], case 12 [26], case 13 [5]

**Table 3:** Summary of the conditions of 35 patients with *F. nucleatum* bacteremia

Characteristics	Age ≥ 40 years	Male	Fever and chills	Had cancer	Survived
n	28	23	16	12	28
%	80.0	65.7	45.7	34.3	80.0

There have so far been no specific recommendations on the antibiotic therapy of infections due to *F. nucleatum*. Three case reports put forward that *F. nucleatum* is resistant to penicillin, amoxicillin, amox-clav [5], and metronidazole [19], but there is no evidence for the 2–6-week treatment [7] recommended by most doctors.

We treated our patient empirically in accordance with the 2016 SSC guidelines, and the subsequent in vitro susceptibility test of the isolate showed that meropenem was effective. Our patient might have died from the complications associated with the infection rather than from failure of antibiotic therapy.

We conclude that as with other abdominal tumors, *F. nucleatum* may also be associated with kidney tumors, and that septic thrombo-embolization and severe sepsis could complicate the post-operative management of such cases.

## Data Availability

The data and materials, including all the clinical data of the patients are included within the article.

## Abbreviations

CT: Computed tomography
CRP: C-reactive protein
PCT: Procalcitonin
SOFA: Sepsis-related Organ Failure Assessment
SSC: Surviving Sepsis Campaign
